# Anticancer Effects of Plasma-Treated Water Solutions from Clinically Approved Infusion Liquids Supplemented with Organic Molecules

**DOI:** 10.1021/acsomega.3c04061

**Published:** 2023-09-01

**Authors:** Valeria Veronico, Sabrina Morelli, Antonella Piscioneri, Roberto Gristina, Michele Casiello, Pietro Favia, Vincenza Armenise, Francesco Fracassi, Loredana De Bartolo, Eloisa Sardella

**Affiliations:** † Department of Chemistry, University of Bari Aldo Moro, via Orabona, 4, 70126 Bari, Italy; ‡ CNR-Institute on Membrane Technology (CNR-ITM), Via Pietro Bucci Cubo, 17/C, 87036 Rende, CS, Italy; § CNR-Institute of Nanotechnology (CNR-NANOTEC), Via Amendola, 122/D, 70124 Bari, Italy

## Abstract

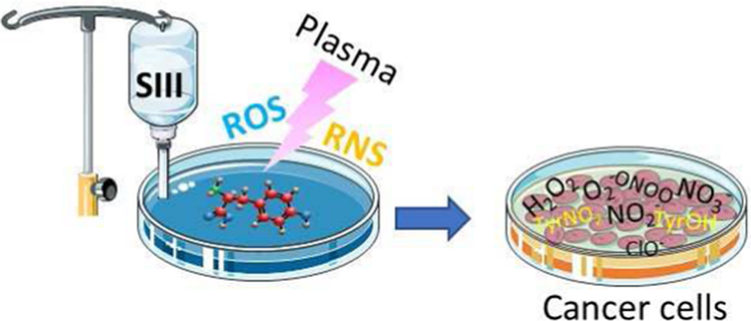

Water solutions treated by cold atmospheric plasmas (CAPs) currently stand out in the field of cancer treatment as sources of exogenous blends of reactive oxygen and nitrogen species (RONS). It is well known that the balance of RONS inside both eukaryotic and prokaryotic cells is directly involved in physiological as well as pathological pathways. Also, organic molecules including phenols could exert promising anticancer effects, mostly attributed to their pro-oxidant ability in vitro and in vivo to generate RONS like O_2_^–^, H_2_O_2_, and a mixture of potentially cytotoxic compounds. By our vision of combining the efficacy of plasma-produced RONS and the use of organic molecules, we could synergistically attack cancer cells; yet, so far, this combination, to the best of our knowledge, has been completely unexplored. In this study, l-tyrosine, an amino acid with a phenolic side chain, is added to a physiological solution, often used in clinical practice (SIII) to be exposed to plasma. The efficacy of the gas plasma-oxidized SIII solution, containing tyrosine, was evaluated on four cancer cell lines selected from among tumors with poor prognosis (SHSY-5Y, MCF-7, HT-29, and SW-480). The aim was to induce tumor toxicity and trigger apoptosis pathways. The results clearly indicate that the plasma-treated water solution (PTWS) reduced cell viability and oxygen uptake due to an increase in intracellular ROS levels and activation of apoptosis pathways in all investigated cancer cells, which may be related to the activation of the mitochondrial-mediated and p-JNK/caspase-3 signaling pathways. This research offers improved knowledge about the physiological mechanisms underlying cancer treatment and a valid method to set up a prompt, adequate, and effective cancer treatment in the clinic.

## 1 Introduction

Over the years, a great deal of academic and clinical research has been done to understand, control, and contain cancer. So far it is well known that reactive oxygen and nitrogen species (RONS) are simultaneously cancer promoters and antagonists, with there being a fine border between the effects of cell exposure to these two species.^1^

Concerning this last aspect, cold atmospheric plasmas (CAPs) have recently gained attention in the field of cancer treatment due to their ability to easily generate RONS at room temperature and tune their dose for specific goals. When O_2_ and N_2_ mixtures are used, CAP consists of gaseous blends of primary RONS^2^ that can be delivered to living matter (cells, tissue, or physiological liquids) directly or indirectly, through aqueous solutions and/or hydrogels as intermediate vehicles.^3−5^ Liquids exposed to plasma and enriched with RONS, defined as plasma-treated water solutions (PTWS), proved to have the same efficacy as that of direct CAP treatment in the eradication of several cancer lines derived from poor-prognosis tumors, due to their resistance to conventional cancer therapies (e.g., melanoma,^6^ pancreatic,^6−8^ colorectal,^9,10^ osteosarcoma,^11^ and ovarian cancer cells^12−14^). The anticancer effects of PTWS are mainly linked to their enrichment with long-lived secondary RONS like hydrogen peroxide (H_2_O_2_), nitrate (NO_3_^–^), and nitrite (NO_2_^–^) ions among others^15^ delivered to the cells. Moreover, it is known that PTWS are more effective than mock solutions prepared by mixing different typologies and concentrations of stable RONS like H_2_O_2_ and NO_2_^–^ when organic molecules are present in the solutions, like in the case of plasma-treated cell culture media.^16^

Phenols and polyphenols are a category of organic molecules whose role as double-edge effectors in cancer treatment has recently been confirmed.^17^ Besides the well-known chemo-preventive effects as antioxidants,^18,19^ in some studies phenols appeared to be also capable of promoting pro-oxidant and cytotoxic effects.^20^ For this reason, phenols are investigated as potential anticancer drugs alone^20^ or in combination with chemotherapies.^20−22^ Tea catechins and related polyphenols were found to inhibit matrix metalloproteinase, which is intimately associated with tumor invasion and metastasis.^23^ The pro-oxidant effects of phenols rely on their oxidation, which leads to the production of O_2_^–^, H_2_O_2_, and a complex mixture of semiquinones and quinines, which are potentially cytotoxic.^17^

We hypothesize that the anticancer effects of PTWS could be improved by the addition of phenols to the liquids before the plasma treatment; indeed, the presence of phenols, along with their derivatives generated after the treatment, may synergistically boost the cytotoxic action of PTWS by increasing the formation of secondary RONS. The combined action of PTWS and phenols against cancer has not been explored yet, to the best of our knowledge, and could represent a valuable approach to treat poor-prognosis tumors that would be resistant to the oxidative stress induced by PTWS alone.

Based on these considerations, our idea was to combine the pro-oxidant effect of l-tyrosine, a natural phenolic amino acid normally present in living organisms, with the oxidative environment of PTWS. As shown in literature, tyrosine and its derivatives in living systems can act, per se, by limiting the progression of tumors.^24^ Thus, we suppose that the production of PTWS containing tyrosine can exacerbate the oxidative stress of cancer cells due to the combined action of exogenous RONS and O- and N-containing plasma-produced products of tyrosine. In fact, in mammals, amino-acid derivatives contribute to epigenetic regulation and immune responses linked to tumorigenesis and metastasis.^24^ Indeed, in vivo redox reactions of tyrosine play a key role in many biological processes, including water oxidation and DNA synthesis.^25^

Most of the in vitro investigations of PTWS containing organic molecules shown in the literature deal with liquids not medically approved, like cell culture media, thus limiting a real clinical translation. To this purpose, for our experiments we started from a water-based injectable preparation, namely SIII solution, which belongs to the category of intravenous electrolytic solutions. SIII is a medical-grade physiological solution used to replenish electrolytes in patients and we enriched such solutions with l-tyrosine before plasma treatment.

The potential anticancer effect of PTWS containing tyrosine was evaluated on four cancer cell lines, SHSY-5Y (neuroblastoma), MCF-7 (breast), HT-29, and SW-480 (colorectal), which are all representative of tumors with poor prognosis. To mimic the cytoarchitecture of cancer, the cells were cultured within a biomimetic membrane system and incubated with different PTWS to assess their ability to counteract the growth and survival of tumor cells.

## 2 Materials and Methods

### 2.1 CAP Treatment of Electrolyte Rehydrating III Solution

**Figure 1:**
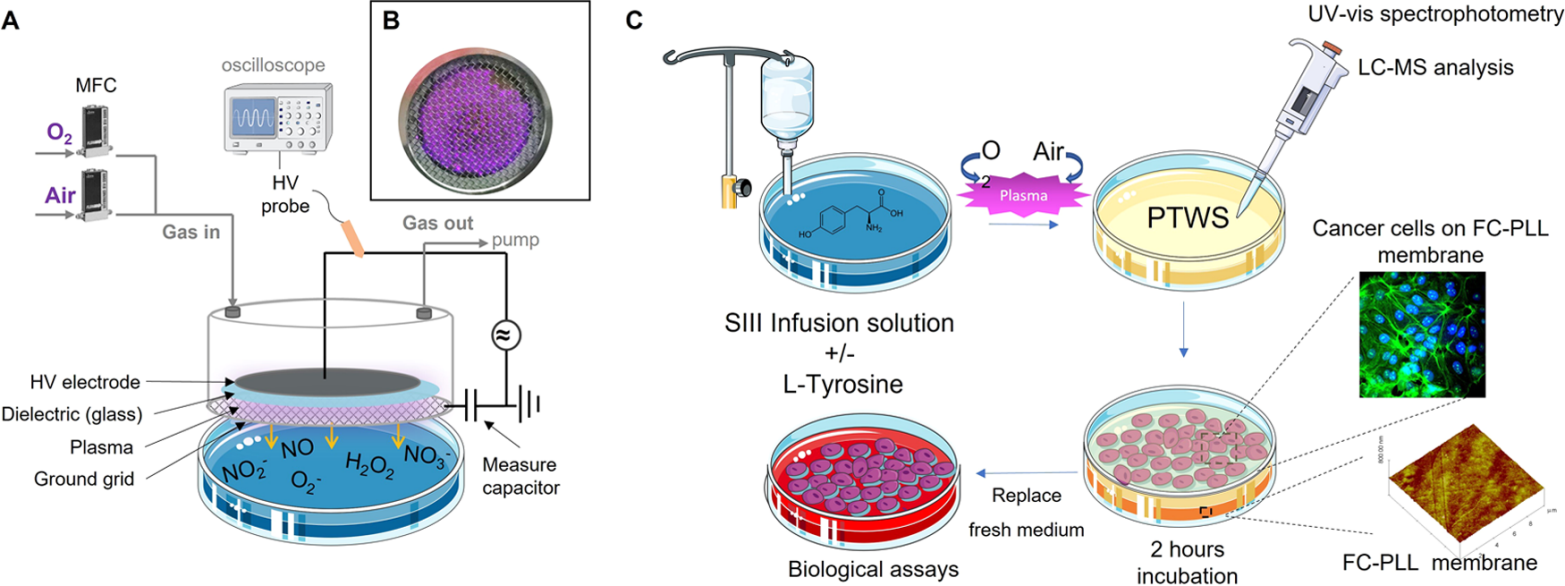
Experimental apparatus and scheme of the research. (A) Schematic overview of the DBD plasma source; (B) image of the discharge unit with plasma ignited; (C) scheme of the production of PTWS and their chemical/biological characterization. A confocal microscope image of cancer cells and an AFM image of the FC-PLL membrane used as substrate for cell seeding are shown on the right side of the scheme.

Electrolyte Rehydrating III solution (SIII, Fresenius Kabi) is a water-based injectable preparation containing sodium, calcium, magnesium, and potassium chlorides, sodium acetate, and sodium citrate. SIII aliquots with and without the addition of different concentrations of amino-acid l-tyrosine (Sigma-Aldrich) have been exposed to a dielectric barrier discharge (DBD) plasma source. A schematic overview of the experimental apparatus and the approach used for the experiments, as well as the picture of the glow of the DBD are, respectively, reported in Figure 1A,B.

A detailed description of the DBD source is provided in ref (26). To plasma-treat the liquids, commercial TPP Petri dishes (57 mm diameter, Techno Plastic Products, Trasadingen, CH) were filled with 2 mL of the SIII solution and housed below the source using a slot on the flow unit. In this way, the liquid was exposed to a volume discharge as wide as the HV electrode (Figure 1B) and treated with the plasma effluents, which diffused through the ground mesh (3 mm far from the liquid surface). Before igniting the plasma, the gap between the liquid and the discharge was purged with the gas feed for 1 min. All discharges were ignited at a constant gas flow rate (0.5 L·min^–1^), field frequency (6 kHz), and applied voltage (13.5 kV), and pulsed with a 25% duty cycle (D.C.; 25 ms plasma on, ton) over a period (*t* = *t*_on_ + *t*_off_) of 100 ms. The treatment time was varied from 45 to 300 s. Pure O_2_ (air liquid, 99.999%) and synthetic air (air liquid, 99.999%) were alternatively used as the gas feed of the DBD source. Depending on the feed used, PTWS were named SIII-tyr in case of untreated SIII solution added with 300 mg/L of tyrosine, and air-DBD and O_2_-DBD in case of SIII-tyr solutions plasma-treated in synthetic air and O_2_, respectively. The pH variation of the liquids was monitored with a pH meter (Hanna instruments, HI8424) before and after plasma treatments.

### 2.2 Detection of H_2_O_2_ and NO_2_^–^

Both H_2_O_2_ and NO_2_^–^ were quantified in PTWS with colorimetric assays and UV–Vis absorbance measurements soon after the DBD treatment (Figure 1C). The Griess assay was used to detect NO_2_^–^ ions by adding 13 mg of reagents from a ready-to-use kit (Merck Millipore) to 1 mL of the samples. A copper-neocuproine assay was used to detect H_2_O_2_ as follows: 100 μL of CuSO_4_ (CuSO_4_·5H_2_O, Sigma-Aldrich) 0.012 M in water, 100 μL of phosphate buffer 0.01 M at pH 7.00 in water (Na_2_HPO_4_ cat. no. 30427, Riedel-de Haën; NaH_2_PO_4_·H_2_O, Carlo Erba), and 100 μL of neocuproine (Sigma-Aldrich) 0.06 M in methanol were sequentially added to 700 μL of the liquid sample and left to react for 10 min. UV–Vis absorbance measurements were performed with a Cary 60 UV–Vis spectrophotometer (Agilent, Santa Clara, CA) in the spectral range of 200–800 nm with a 5 nm resolution. Disposable plastic cuvettes (Bio-Rad, Hercules, CA) with a semi-micro volume of 1 mL and 10 mm optical length were used. Both assays were specifically validated in the case of PTWS as shown in our previous paper.^27^ To study how the PTWS chemical composition changes over time, chemical analyses were carried out immediately after plasma treatment and after up to 15 days of storage at 4 and 25 °C. These results were compared with those obtained from a mock solution prepared by adding H_2_O_2_ to SIII solution (80 μM).

### 2.3 High-Resolution Mass Spectrometry

Mass spectra were obtained in the electrospray ionization (ESI) positive- and negative-ion modes using an LC-MS-IT-TOF (Shimadzu) mass spectrometer equipped with a binary pump (NexeraXR, LC-20ADxr), an auto-sampling system (NexeraXR, SIL-20ADxr), and a photodiode array (SPD-M20A) detector. CDL and heat block temperature were set at 250 °C, and the mass screening was done in the range 100–500 *m*/*z*. A detector voltage of 1.7 kV and a flow rate of 1.5 L/min of the nebulizing gas (N_2_) were used for the purpose.

A volume of 2 μL of the sample was directly infused into the spectrometer with an acid mobile phase (i.e., a mixture of 4-fold v/v of methanol and 3-fold v/v of acetonitrile and 0.1% v/v of formic acid) to improve the sensitivity of detection. The solutions were electro-sprayed at a flow rate of 0.3 mL·min^–1^. Qualitative analysis was carried out by comparing the exact masses with the isotopic distribution, and through a study of the fragmentation pattern of MS^2^ when available. The LC-MS solution (V3.80.410, Shimadzu) software was used for data acquisition and processing; the characterization was processed with the Shimadzu LabSolutions Lite V5.82 software package (Formula Predict and Accurate Mass Calculator).

### 2.4 Cell Culture and Treatments

Four different cancer cell lines were cultured in a membrane system consisting of a gas-permeable (CO_2_, O_2_ and H_2_O vapor) fluorocarbon membrane, in flat configuration (FC, In Vitro Systems & Services, Germany). FC membranes were functionalized with a coating of poly-l-lysine (PLL, MW 30 000–70 000, Sigma-Aldrich) [40 μg/cm^2^] to improve cell adhesion and growth, as described in ref^28^

The SH-SY5Y human neuroblastoma cell line (ICLC-IST, Genova, Italy) was cultured in a 1:1 mixture of Ham’s F12 (Invitrogen, Milan, Italy) and Minimum Essential Eagle’s medium (EMEM) supplemented with 10% (v/v) heat-inactivated FBS (Euroclone), 2 mM glutamine, and 100 μg/mL penicillin/streptomycin.^29^

The MCF-7 human breast cancer cell line (ICLC-IST, Genova, Italy) was grown in Dulbecco’s Modified Eagle Medium (DMEM) with 1 g/L of glucose GIBCO, Thermo Fisher Scientific, supplemented with 10% (v/v) FBS, 2 mM glutamine, and 1% penicillin/streptomycin.

The HT-29 and SW-480 human colorectal adenocarcinoma cell lines (ATCC) were cultured in DMEM with 4.5 g/L of glucose (GIBCO, Thermo Fisher Scientific), supplemented with 10% (v/v) FBS, 2 mM glutamine, and 1% (v/v) penicillin/streptomycin.

Cells were maintained at 37 °C, 5% CO_2_ in a humidified incubator in 75 cm^2^ flasks (PBI International, Milan, Italy). Subconfluent SHSY-5Y, MCF-7, HT-29, and SW-480 cells were treated with trypsin, centrifuged, and seeded in a FC-PLL membrane system at 3.5 × 10^4^ cells/cm^2^ in their corresponding medium. After 24 h, the medium was replaced with PTWS. Cells were exposed in triplicate to SIII-tyr, air-DBD, and O_2_-DBD. As positive control (CNTR), cell lines incubated in their media were used. After 2 h of incubation, all solutions were replaced with fresh complete medium and then incubated at 37 °C for 24, 48, and 72 h.

### 2.5 Viability Assay

Cell viability was evaluated with the trypan blue exclusion test, based on the so-called nuclear-exclusion principle: trypan blue is excluded from viable cells but permeates into dead cells. After 24, 48, and 72 h from the 2-h incubation with different PTWS, cells were detached from the FC-PLL membrane system with a trypsin/EDTA solution (Euroclone) and centrifuged (600 *g*, 5 min, 25 °C). Cell pellets were resuspended in culture media and the total number of cells was determined using a trypan blue stain and a hemocytometer under a light optic microscope (Axio Vert, Zeiss, Germany). Cell viability was expressed as the number of viable cells per cm^2^.

### 2.6 Intracellular ROS Detection

The formation of intracellular ROS was investigated using 2′,7′-dichlorodihydrofluorescein diacetate (H_2_DCF-DA, Sigma-Aldrich), as described in a previous paper.^30^ After diffusing into the cell, this molecule was deacetylated by cellular esterase to a nonfluorescent compound. The compound so produced was later oxidized by ROS into the highly fluorescent 2′,7′-dichlorofluorescein (DCF), whose fluorescence intensity is proportional to the ROS level. After 24, 48 and 72 h from the 2-h exposure to PTWS, tumor cells were loaded with 50 μM H_2_DCF-DA at 37 °C for 30 min. Cells were rinsed twice with Hank’s salt solution and then DCF fluorescence intensity was measured with a laser scanning confocal microscope (LSCM, Fluoview FV300, Olympus, Milan, Italy) using an Ar laser. Quantitative analysis was performed on confocal images of cells by using the Fluoview 5.0 software (Olympus Corporation).

### 2.7 Apoptosis Detection

Apoptosis of cells was investigated by evaluating the mitochondrial membrane potential (MMP) and the activation of specific apoptotic markers by LSCM. The MMP of cancer cells was measured using the fluorescent, lipophilic, cationic dye tetraethyl-benzimidazolyl-carbocyanine iodide (JC-1, Sigma-Aldrich) that accumulates in energized mitochondria. JC-1 forms an aggregate (in healthy mitochondria) with red fluorescence. As the membrane potential decreases, JC-1 becomes monomers, characterized by green fluorescence. The change in the ratio of red to green fluorescence is used as an indicator of the mitochondrial condition.

At 24, 48, and 72 h after the 2-h exposure to PTWS, cells were stained with JC-1 according to a protocol previously reported.^31^ Cell cultures were incubated at 37 °C with JC-1 (5 μg/mL) for 20 min. The fluorescence intensity of JC-1 monomers (green) and aggregates (red) was detected in LSCM using an Ar laser and a He/Ne laser, respectively. Then, the MMP of cells was expressed for each treatment as the JC-1 red/green fluorescence ratio.

The activation and expression of two apoptotic markers, caspase-3 and phosphorylated N-terminal c-Jun protein kinase (p-JKN), were detected by performing immunostaining for LSCM analysis.^32^

At 24, 48, and 72 h after the 2-h treatments with the different solutions, cells were rinsed with PBS and fixed with 4% (wt/v) paraformaldehyde for 15 min, followed by permeabilization and blocking with a solution of 0.3% (v/v) Triton X-100 and 10% (v/v) FBS in PBS for 1 h at 37 °C. The samples were then incubated overnight at 4 °C with rabbit anti-caspase-3 antibody (1:250; BD Franklin Lakes) and mouse anti-phospho-JNK (Thr183/Tyr 185) antibody (1:250; Santa Cruz Biotechnology, CA). Secondary antibodies, Cy2TM-conjugated Affini Pure donkey anti-rabbit IgG and Cy3TM-conjugated Affini Pure donkey anti-mouse IgG (1:500, Jackson ImmunoResearch Europe Ltd.), were added for 1 h at 25 °C (RT). Finally, the cells were counterstained with 200 ng/mL DAPI (Molecular Probes) for nuclear localization. Samples were rinsed, mounted, and observed at LSCM.

Quantitative analysis of apoptotic cells was performed by calculating the percentage of positive cells for each marker (caspase-3 positive nuclei and p-JNK positive nuclei) over the total (DAPI-stained nuclei), counted in different culture conditions.

### 2.8 Metabolic Activity

The metabolic activity of the cells was evaluated by investigating the oxygen uptake rate (OUR). The O_2_ concentration of the culture medium was noninvasively detected with a Sensor Dish Reader (SDR), OxoDish-DW (PreSens Precision Sensing GmbH), as previously reported,^31^ which allows the real-time in situ measurements of dissolved oxygen.

### 2.9 Statistical Analysis and Data Processing

Differences among data were computed with the statistical software GraphPad Prism 6.1. Concerning the RONS concentrations detected in PTWS, a one-way ANOVA was performed with a subsequent Turkey’s Multiple Comparison Test by assuming a normal distribution in the data. Statistical results were shown as the mean value ± standard deviation (SD) of the data. All *p*-values <0.001 were considered statistically significant.

With regard to the biological analysis, the statistical significance of the experimental results was assessed using a one-way ANOVA test followed by a Bonferroni *t*-test (*p* < 0.05) looking at the differences among the PTWS incubation in the same culture time. The results were expressed as mean ± SD from at least three independent experiments in which five samples per treatment were assessed.

## 3 Results and Discussion

### 3.1 Chemical Composition of PT-SIII

**Figure 2:**
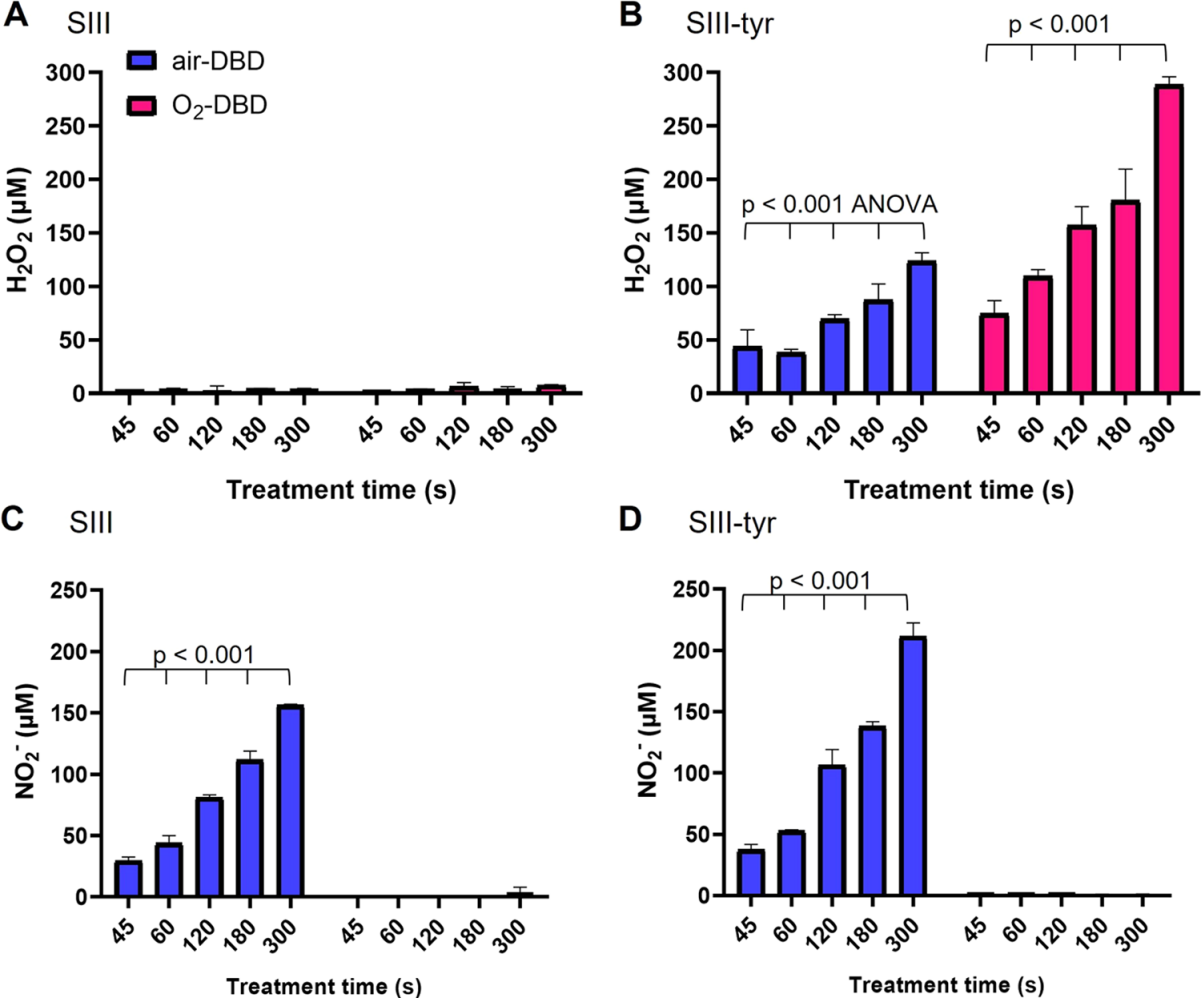
Production of RONS. H_2_O_2_ and NO_2_^–^ concentrations generated in SIII without (A–C) and with the addition of tyrosine (300 mg/L) (B–D), after igniting air- or O_2_-fed DBDs for different treatment times. DBDs were ignited at 13.8 kV, 6 kHz, 25% DC, 0.5 slm flow rate in 2 mL of liquids 3 mm far from the ground electrode.

H_2_O_2_ and NO_2_^–^ have been considered representative, respectively, of the reactive oxygen species (ROS) and reactive nitrogen species (RNS) generated in PTWS, aware that they are only a part of the large variety of RONS produced in water solutions after plasma processing (e.g., O_2_^–^, OH, HOO^–^, NO_2_, NO etc.) along with halogenated compounds (e.g., ClO^–^ etc.). The results of H_2_O_2_ and NO_2_^–^ detection are reported in Figure 2. The data clearly indicate that the presence of l-tyrosine improves the generation of RONS in the plasma-treated (PT) liquids, especially H_2_O_2_. The pH of PT-SIII-tyr remains unaltered around the physiologic one with respect to the untreated SIII-tyr solution irrespective of the plasma treatment used (Figure S1).

It can be noted that H_2_O_2_ in PT-SIII is not present (<10 μM, limit of detection, LOD, of the colorimetric assay),^27^ after both O_2_- and air-fed DBDs, for treatment times up to 300 s (Figure 2A). On the contrary, when l-tyrosine is added (300 mg/L), the generation of H_2_O_2_ occurs, with a trend dependent on the treatment time and on the amount of O_2_ in the feed (Figure 2B). O_2_-fed DBDs produced higher amounts of H_2_O_2_ in PT-SIII-tyr than air-fed DBDs, for the same treatment times (Figure 2B).

The results here reported are in agreement with our recent findings for PTWS from double-distilled water, which demonstrated that the H_2_O_2_ generation in our processes (i.e., plasma not in contact with the liquid, closed chamber of the reaction) is promoted by the oxidation reactions of organic compounds with a phenolic ring, probably triggered by transient ROS like atomic oxygen and O_3_.^26^ Similar results were also obtained by Hefny et al.,^33^ whose results demonstrate that the H_2_O_2_ formation in deionized water is dependent on the presence of phenol in the liquid phase, in the absence of humidity in the plasma phase (i.e., negligible evaporation of water).^33^ H_2_O_2_ production, in our processes, surely involves the oxidation of organic molecules, differently from what is generally assumed in the literature, where OH recombination in the plasma or the liquid is considered the main mechanism.^15,34−39^

NO_2_^–^ generation, however, is only slightly affected by the addition of tyrosine to the SIII solution (PT-SIII-tyr). The trend for NO_2_^–^ in PT-SIII (Figure 2C) and in PT-SIII-tyr (Figure 2D) confirms that NO_2_^–^ ions are formed in both liquids only after air-fed DBD treatments, at concentrations that increase with the treatment time. Conversely, after O_2_-fed-DBD, no nitrite ions could be detected in PT-SIII as well as in PT-SIII-tyr (Figure 2C,D), in agreement with our recent findings.^26^ We have recently demonstrated that the NO_2_^–^ formation in PTWS is prevalently due to the RNS (presumably NO or NO_2_) generated in the plasma. Therefore, the amount of NO_2_^–^ ions in PTWS can be modulated by properly adjusting the proportion between O_2_ and N_2_ in the feed, and other parameters such as applied voltage and treatment time.^26^ Finally, despite the presence of nitrites produced by air-DBD (Figure 2D), indicative of the presence of nitrous acid that produces certain acidification of the PTWS, we did not observe such effect probably due to the ability of SIII components (i.e., citrate and acetate) to act as buffers.

**Figure 3:**
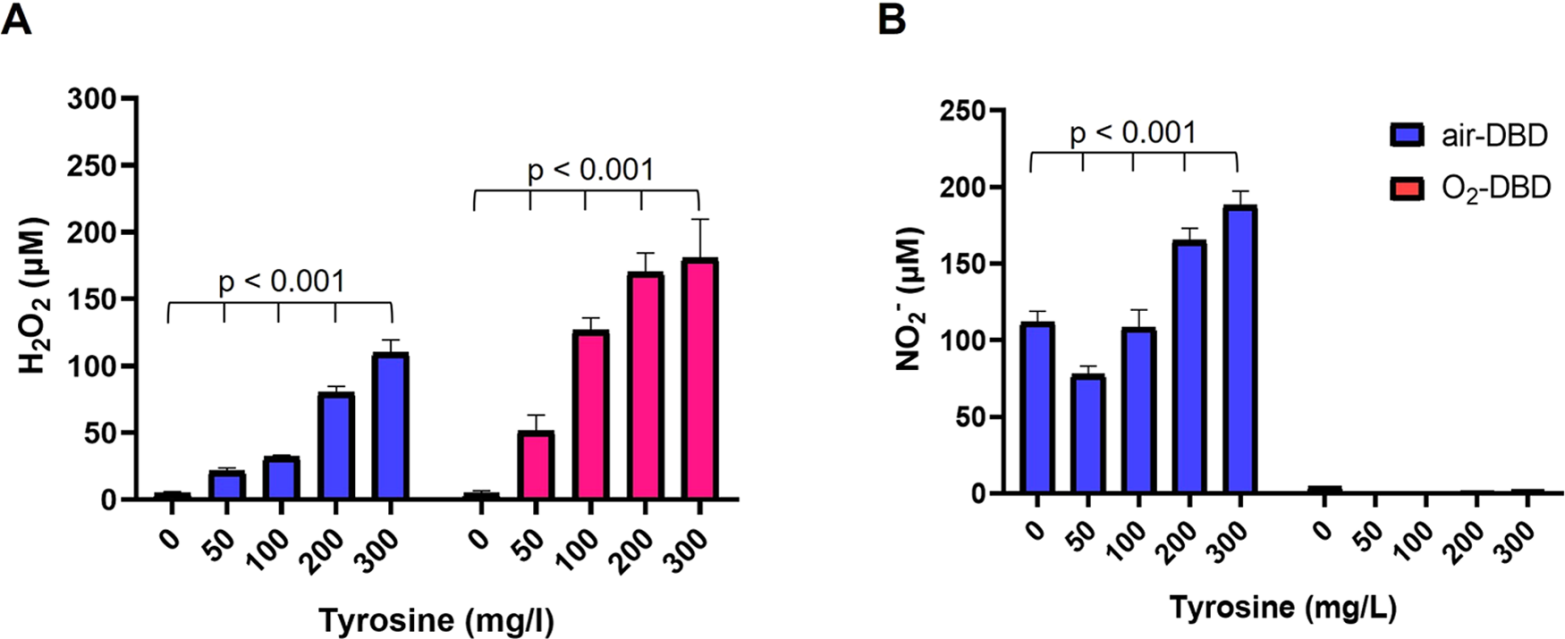
Analysis of RONS in SIII-tyr solutions. Concentration of (A) H_2_O_2_ and (B) NO_2_ generated in SIII at increasing concentrations (0–300 mg/L) of added tyrosine, after 3 min of air- or O_2_-fed DBD (13.8 kV, 6 kHz, 25% DC, 0.5 slm feed flow rate, 2 mL liquids 3 mm far from the ground electrode).

To better elucidate the pro-oxidant activity of l-tyrosine and its contribution to RONS generation, we have monitored the H_2_O_2_ and NO_2_ generation in SIII at increasing concentrations of l-tyrosine (0–300 mg/L), after 3 min of air- or O_2_-fed DBD. The results reported in Figure 3 demonstrate that the amounts of H_2_O_2_ and NO_2_^–^ and, presumably, of other secondary RONS produced in the PTWS, are significantly dependent on l-tyrosine concentration.

This trend is more pronounced in the case of H_2_O_2_ with respect to NO_2_^–^ (Figure 3A vs Figure 3B). It is also important to highlight that without tyrosine in the liquid (i.e., 0 mg/L of Figure 3A) no H_2_O_2_ is produced. We can conclude that, by varying the concentration of organic molecules with phenolic moieties added to the liquids, the generation of RONS can be finely modulated in PTWS.

To assess if and how the organic molecules present in SIII-tyr are involved in the plasma treatment and identify their derivatives, Hi-Res mass spectrometry was carried out on untreated liquids and PTWS.

**Figure 4:**
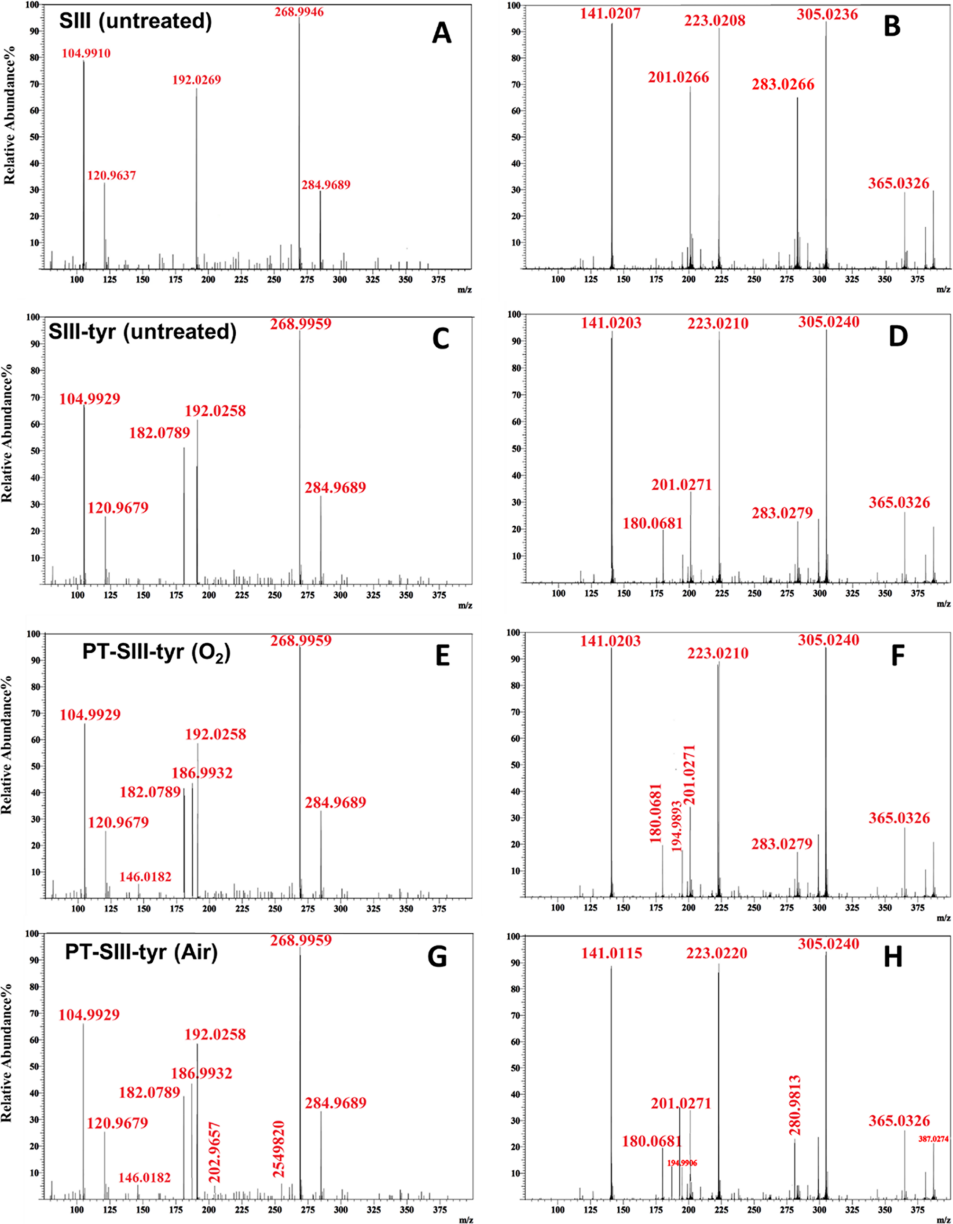
LC-MS of SIII and SIII-tyr solutions. Positive (A, C, E, G) and negative (B, D, F, H) mode spectra acquired for (A, B) untreated SIII; (C, D) untreated SIII-tyr; (E, F) SIII-tyr treated with 45 s of O_2_ plasma; and (G, H) SIII-tyr treated with 3 min air plasma (13.8 kV, 6 kHz, 25% DC, 0.5 slm feed flow rate, 2 mL liquids 3 mm far from the ground electrode).

The positive and negative mode mass spectra acquired on SIII and SIII-tyr solutions before and after oxygen and air plasma treatment are reported in Figure 4.

In SIII and SIII-tyr untreated solutions, the presence of the cation signals of citric acid (Citr^+^ 192.0269 *m*/*z*) and protonated tyrosine (Tyr + H^+^ 182.0789 *m*/*z*), typical of some of the main products of the solutions, is evident. After plasma treatment only slight changes are revealed in the positive ion spectra (Figure 4E,G): both plasma treatments produce the 146.0169 *m*/*z* peak (citrate–CO_2_); the O_2_ plasma also produces a peak at 186.9930 *m*/*z*, probably due to a calcium complex of the fragment at 146.0169 *m*/*z*.

The analysis of negative ions spectra reveals new signals at 194.9892 and 280.9813 *m*/*z* after both DBDs: the former can be attributed to the degradation of sodium citrate (C_6_H_4_O_6_Na), while the latter should be due to the formation of a nitro derivative of citrate (C_6_H_5_N_2_O_11_). It is worth noting that the co-presence of various cations and anions in the SIII solution renders the analysis very hard due to the generation of several adducts that probably hide those derived from the reaction of tyrosine. In fact, by analyzing plasma-treated l-Tyrosine solutions in double-distilled water, generated in the same conditions shown in this paper, it is possible to clearly see the products of nitration (Tyr + N + 3O–H)^−^ and oxidation (Tyr + 3O–H)^−^ of tyrosine after the two different DBDs (Figure S3). All of the organic derivatives produced in PT-SIII-tyr solutions including the ones derived from citrate and from tyrosine could actively contribute to produce an effect on cells.

### 3.2 Stability of PTWS Produced from SIII Solution and l-Tyrosine

**Figure 5:**
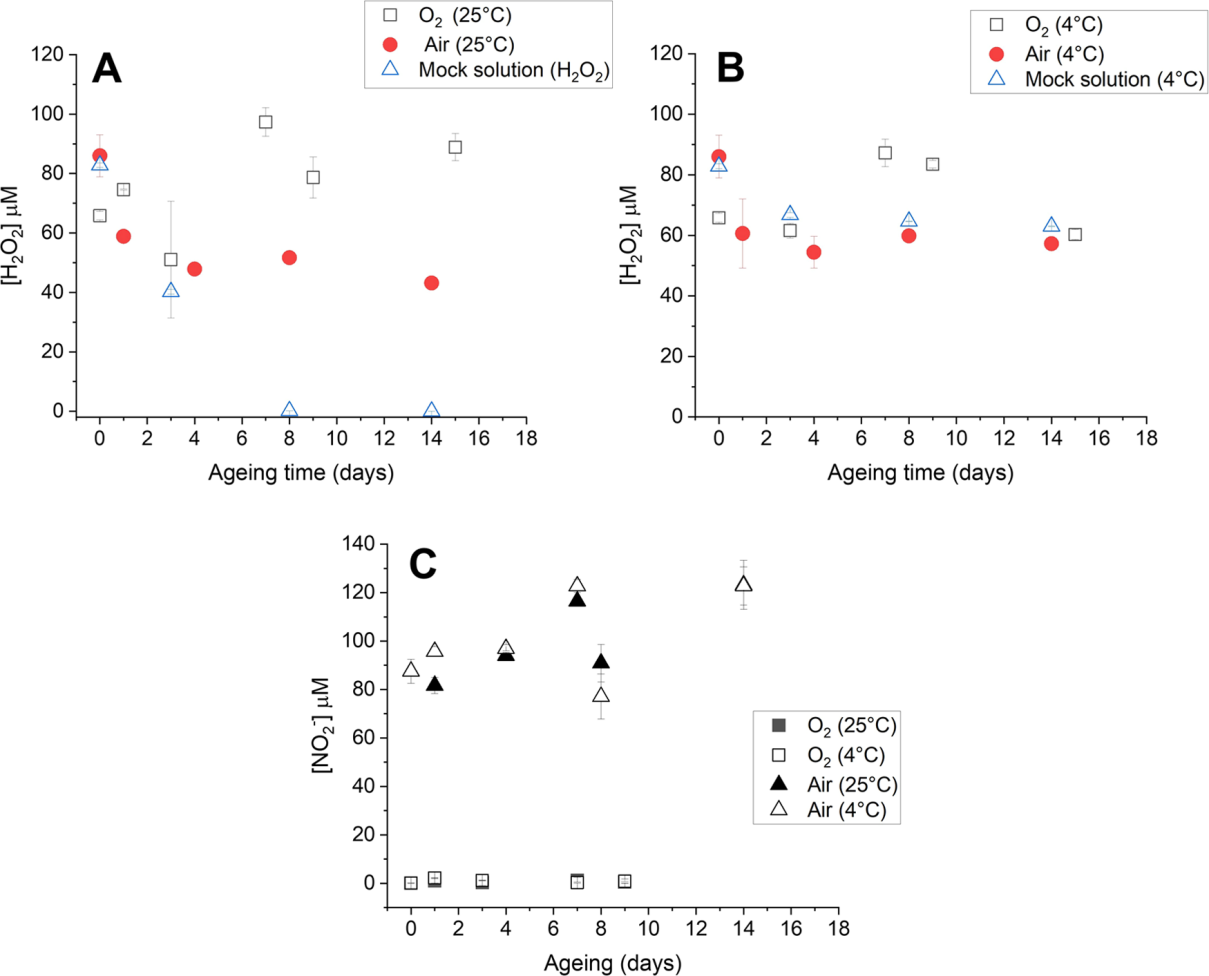
Aging of PTWS and mock solutions in different storage environments. Concentration of H_2_O_2_ at (A) 25 °C and (B) 4 °C and (C) concentration of nitrites at different storage temperatures. PTWS obtained with 3 min air- or O_2_-DBD (13.8 kV, 6 kHz, 25% DC, 0.5 slm flow rate on 2 mL of liquids 3 mm far from the ground electrode) were compared with a mock solution obtained by dissolving H_2_O_2_ in SIII + tyr solution with a final concentration of 80 μM.

A study of the aging of PTWS was carried out to assess the potential for future application of these solutions in biomedical field. The concentrations of H_2_O_2_ and NO_2_^–^ were measured before and after storage at 25 or 4 °C, as reported in Figure 5. Considering that H_2_O_2_ is much more reactive than nitrites, and with the hypothesis that H_2_O_2_ is overproduced by the presence of l-Tyrosine, the aging trends were compared with those of a mock solution containing a concentration of H_2_O_2_ (80 μM) similar to that detected in PTWS. It is possible to observe that, in all PTWS produced, the concentration of nitrites remains stable with time, due to their low reactivity. H_2_O_2_ remains stable in mock solutions only when it is stored at 4 °C, but it is rapidly consumed within the first 8 days when stored at room temperature, differently from PTWS, where the presence of H_2_O_2_ was revealed also after several days of storage. These findings confirm that the reactions involving tyrosine concur to the formation of secondary H_2_O_2_.

The LC-MS analysis of 2-week-aged PT-SIII-tyr solutions (Figure S2) at 25 °C does not reveal significant changes in chemical composition with respect to fresh samples, confirming the stability of the organic component of the solution with time.

### 3.3 Anticancer Efficacy of PTWS

A first preliminary cell culture experiment aimed at verifying the importance of tyrosine in SIII-treated solutions has been performed on two cancer-derived cell lines, HT-29 and SH-SY5Y. The results confirm that the combination of plasma-produced RONS species and tyrosine is effective against both cancer cells by achieving a clear reduction in cell growth (Figures S4 and S5), while treating only with the SIII solution produces a negligible effect on cells (small clusters).

To explore the combined anticancer effect of plasma treatment and l-tyrosine, two different PT-SIII-tyr solutions were chosen: SIII solution with tyrosine (300 mg/L) after a 3-min air-DBD and 45 s O_2_-DBD, respectively. These conditions led to the formation of similar amounts of H_2_O_2_ (75–87 μM) but different concentrations of NO_2_^–^ (2.2–138 μM), as shown in Table 1. As H_2_O_2_ and NO_2_^–^ are representative of all secondary RONS, this approach allowed us to separate the potential anticancer contribution of ROS and RNS delivered by PTWS, including some O- and N-derivatives of organic molecules present in the solutions, whose role cannot be excluded.

**Table 1:** Analysis of RONS in PTWS Selected for In Vitro Biological Experiments^a^

sample name	plasma treatment	H_2_O_2_	NO_2_^–^
SIII-tyr	none	<LOD	<LOD
O_2_-DBD	O_2_-fed-DBD, 45 s on SIII-tyr (300 mg/L tyr)	75 ± 12 μM	2.2 ± 0.2 μM
air-DBD	air-fed-DBD, 3 min on SIII-tyr (300 mg/L tyr)	87 ± 14 μM	138 ± 4 μM

a Concentrations of H_2_O_2_ and NO_2_^–^ detected in SIII with addition of 300 mg/L l-tyrosine after DBD treatments (13.5 kV, 6 kHz, 25% DC, 0.5 slm feed flow rate, 2 mL liquid, 3 mm far from the discharge) fed with air and O_2_.

To realize a reliable in vitro model system, SHSY-5Y, MCF-7, HT-29, and SW-480 cancer cells were cultured within a biomimetic membrane platform in four different liquids: (i) the culture medium (CNTR), used as control; (ii) the untreated SIII-tyr solution; (iii) SIII-tyr treated with 3 -min air DBD (air-DBD); and (iv) SIII-tyr treated with 45 s O_2_-DBD (O_2_-DBD). After 2 h of incubation, all of the liquids were replaced with fresh complete culture medium. The anticancer effect was evaluated at the time frames of 24, 48, and 72 h after the end of PTWS incubation; cell viability, oxidative, and apoptosis-based analyses were performed to determine whether the incubation in PTWS could effectively affect the behavior of tumor cells.

#### 3.3.1 Cytotoxic Effect of PTWS in Cancer Cells

At first, the cytotoxic effects of each PTWS were assessed by investigating their efficacy to downregulate tumor growth, by counting the number of viable cancer cells 24, 48, and 72 h after the 2-h incubation in the different PTWS.

**Figure 6:**
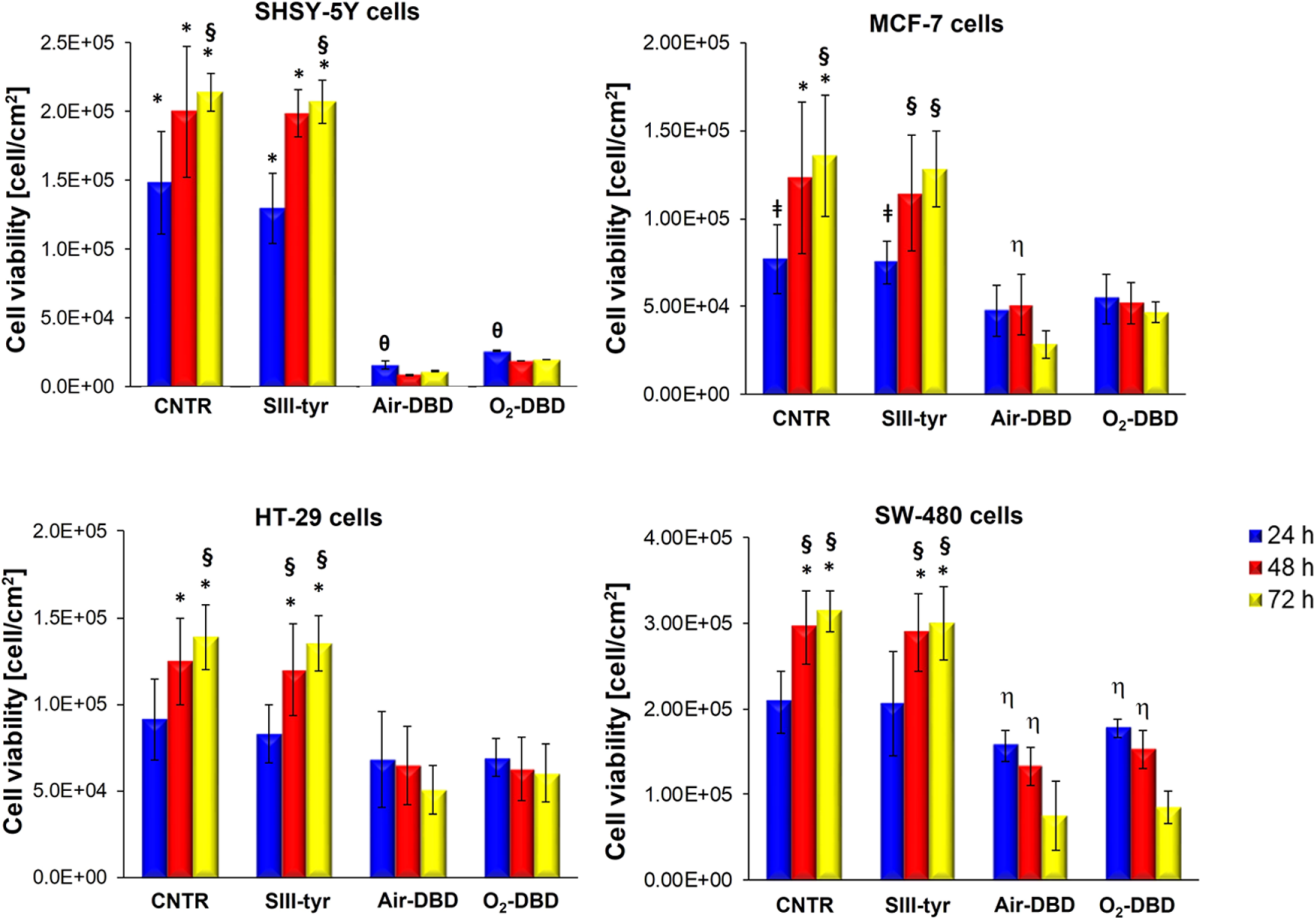
Antiproliferative effect of PTWS in cancer cells. Cell viability after 24, 48, and 72 h of 2-h incubation in SIII-tyr, air-DBD, and O_2_-DBD. Data statistically significant according to ANOVA followed by Bonferroni *t*-test (*p* < 0.05). * vs air-DBD and O_2_-DBD at the same culture time; ^§^ vs 24 h for the same treatment; ^θ^ vs 48 and 72 h for the same treatment; ^⧧^ vs air-DBD at the same culture time; ^η ^vs 72 h for the same treatment.

As reported in Figure 6, incubation with SIII-tyr (not plasma treated) did not affect the viability of all investigated cancer cells, since the values are close to the control at all of the considered culture times. This demonstrates that the infusion solution enriched with l-tyrosine had no cytotoxic effects by itself.

On the contrary, the incubation to the PTWS (air-DBD and O_2_-DBD) influenced the cell viability, with cell type-dependent effects.

In SH-SY5Y human neuroblastoma cells, both PT solutions had a strong cytotoxic effect, causing cell death. Indeed, after 24 h, air-DBD and O_2_-DBD significantly decreased the number of viable cells to values of 1.6 × 10^4^ cell/cm^2^ and 2.6 × 10^4^ cell/cm^2^, respectively, indicating an inhibition of about 90% of cell proliferation with respect to the control.

Different results were observed for the other cell types. Both air-DBD and O_2_-DBD reduced the exponential growth of tumor cells in a time-dependent manner. Air-DBD solutions displayed an interesting relevant cytotoxic activity (after 72 h) toward MCF-7 breast and SW-480 colorectal cancer cells, strongly decreasing the percentage of cell viability. The number of viable SW-480 cells decreased significantly after 72 h from 3.1 × 10^5^ (control) to 7.5 × 10^4^ cells/cm^2^ upon treatment with air-DBD solution. A similar trend was observed for the MCF-7 cells treated with the same solution, with 80% reduction of viable cells compared to the control. PTWS showed also a strong cytotoxic effect toward HT-29 cells, with more than 60% of the treated cells being dead after 72 h.

Relevant to note, both air-DBD and O_2_-DBD solutions showed a strong antiproliferative effect in all types of cancer cells analyzed in this study.

#### 3.3.2 PTWS Display Pro-Oxidant Effects in Cancer Cells

To better ascertain the cytotoxic effects of PTWS that might pass unnoticed in the cell viability assays, further investigation was performed with the final goal of highlighting their anticancer potential.

Since most anticancer drugs work by causing oxidative stress in tumor cells, and excessive intracellular ROS accumulation can lead to irreversible cell damage and apoptosis, we monitored intracellular ROS formation with the H_2_DCF-DA probe to check whether PTWS could cause cytotoxicity by increasing the oxidant status levels in the cells.

Since PTWS completely suppresses SHSY-5Y cell viability, the next investigation dealt with the other three types of cancer cells (MCF-7, HT-29, and SW-480).

**Figure 7:**
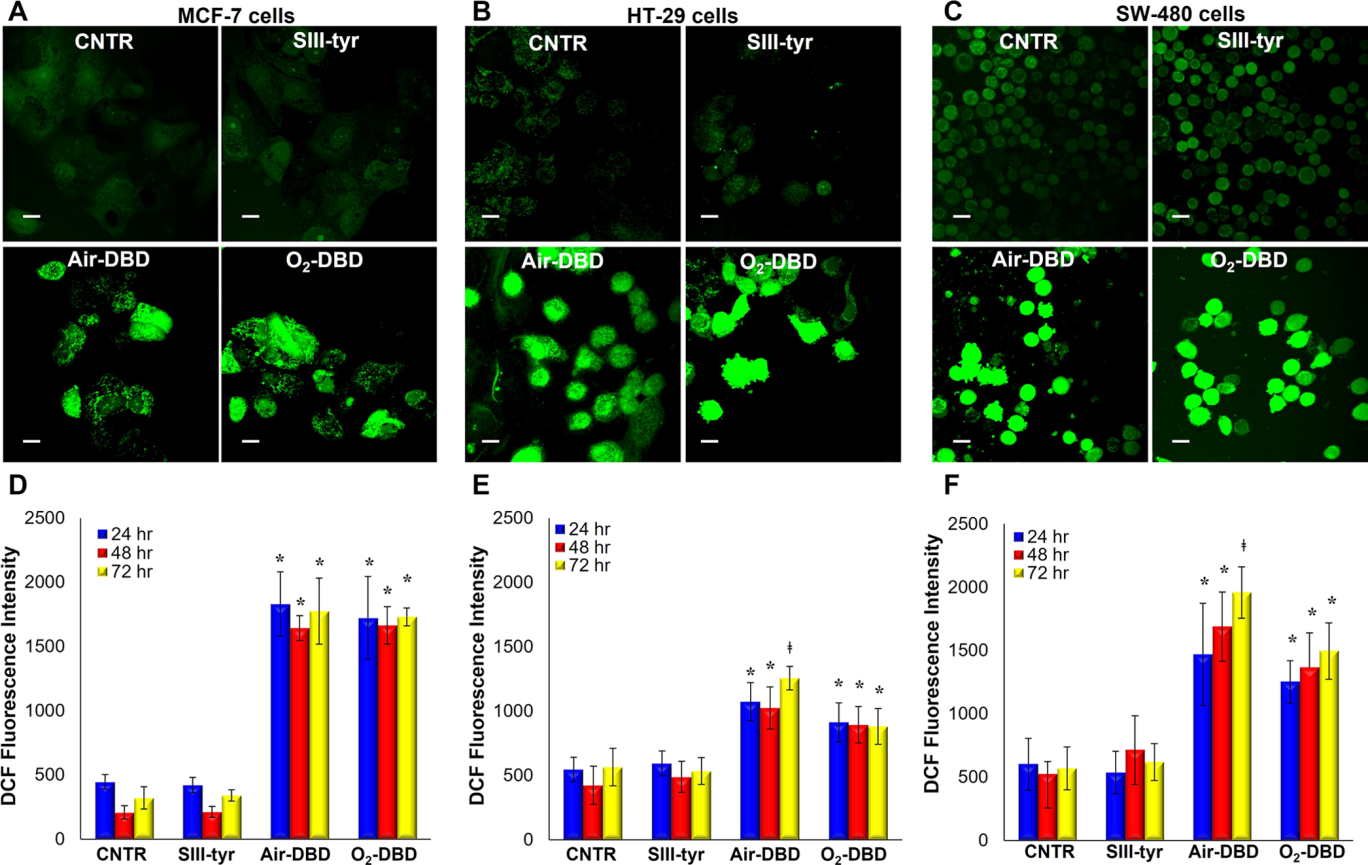
Pro-oxidant effect of PTWS. (A–C) Confocal laser micrographs of the DCF fluorescence signal in MCF-7 (A), HT-29 (B), and SW-480 (C) cancer cells 72 h after 2-h incubation in different solutions with respect to cells grown on untreated medium used as control (CNTR). Scale bar: 20 μm. (D–F) Quantitative analysis of the DCF fluorescence intensity produced in MCF-7 (D), HT-29 (E), and SW-480 (F) tumor cells 24, 48, and 72 h after 2-h incubation in different solutions with respect to cells grown on untreated medium used as control (CNTR). Data statistically significant according to one-way ANOVA followed by Bonferroni *t*-test (*p* < 0.05). *vs CNTR and SIII-tyr at the same culture time; ^⧧^vs all treatments at the same culture time.

Interestingly, the LSCM micrographs reported in Figure 7A–C showed that both PTWS led to a massive production of ROS in these cancer cells, as evidenced by the bright green DCF fluorescence compared with the respective controls. The treatment with SIII-tyr did not provide any increase in DCF fluorescence intensity, and thus in intracellular ROS formation, in all cancer cells. These observations provide further evidence of the nontoxic effect of the untreated SIII-tyr solution, similarly to what was observed after the evaluation of cell viability. We can therefore state that the phenol auto-oxidation of tyrosine is not enough to promote the anticancer properties of such solutions and that the plasma treatment is needed to trigger pro-oxidant effects.

A quantitative analysis of the DCF fluorescence intensity, which is directly linked to ROS production and accumulation, was performed to better highlight the differences between the cancer cells (Figure 7D–F).

In MCF-7 cells (Figure 7D), the ROS levels increased significantly in the presence of both PT-SIII-tyr solutions (Air- and O_2_-DBD), which induced similar values of DCF fluorescence intensity, thereby indicating the induction of a similar pro-oxidant effect of the two different plasma-treated solutions. In both cases, the values of intracellular ROS production are 3-fold higher than those of the control.

Concerning the two colorectal cancer cell lines, HT-29 and SW-480, the two plasma-treated solutions differently affected the intracellular ROS production. In HT-29 (Figure 7E) and SW-480 cells (Figure 7F) exposed to the air-DBD solution, respectively, the production of ROS 72 h after the exposure became 2- and 4-fold higher than that of the control. These values were significantly higher than the O_2_-DBD-induced toxicity at any culture time, in both colorectal cancer cells. A significant increase of ROS production, 1.5- and 1-fold higher than the control, was measured in HT-29 and SW-480 cells, respectively, for the O_2_-DBD solution.

These findings indicate that both PTWS displayed a high pro-oxidant activity toward cancer cells. The increase in intracellular ROS formation agrees with several reports demonstrating that chemotherapeutics exert their anticancer effect by enhancing ROS production. Cisplatin, for example, can kill tumor cells by promoting excessive accumulation of ROS.^39^ Recently, it has been found that an increase in ROS levels in breast cancer and human multiple myeloma can promote tumor cell death and increase the sensitivity to antitumor therapies.^40^

#### 3.3.3 PTWS-Enhanced Apoptosis of Cancer Cells

Pro-oxidant conditions are strictly related to the antitumor mechanisms, being one of the crucial steps that triggers the apoptotic cascade inducing the regression of the tumor. Indeed, high levels of ROS, as those found in all cell lines exposed to PTWS in this study, can trigger apoptotic cell death. Thus, we assessed the potential apoptotic effect of PTWS in cancer cells by investigating the changes in mitochondrial membrane integrity, reported to be one of the early events in apoptosis.^41^ The mitochondria membrane potential (MMP) changes resulting from PTWS treatment were evaluated by means of the membrane-permeant JC-1 probe, widely used in apoptosis studies to monitor mitochondrial health. In healthy cells, JC-1 aggregates in the mitochondria due to the high MMP (hyperpolarization) and yields a red emission. In apoptotic cells, JC-1 exists in monomeric form and stains the cytosol green due to the loss of MMP (depolarization). The mitochondrial depolarization is thus indicated by a decrease in the red/green fluorescence intensity ratio.

**Figure 8:**
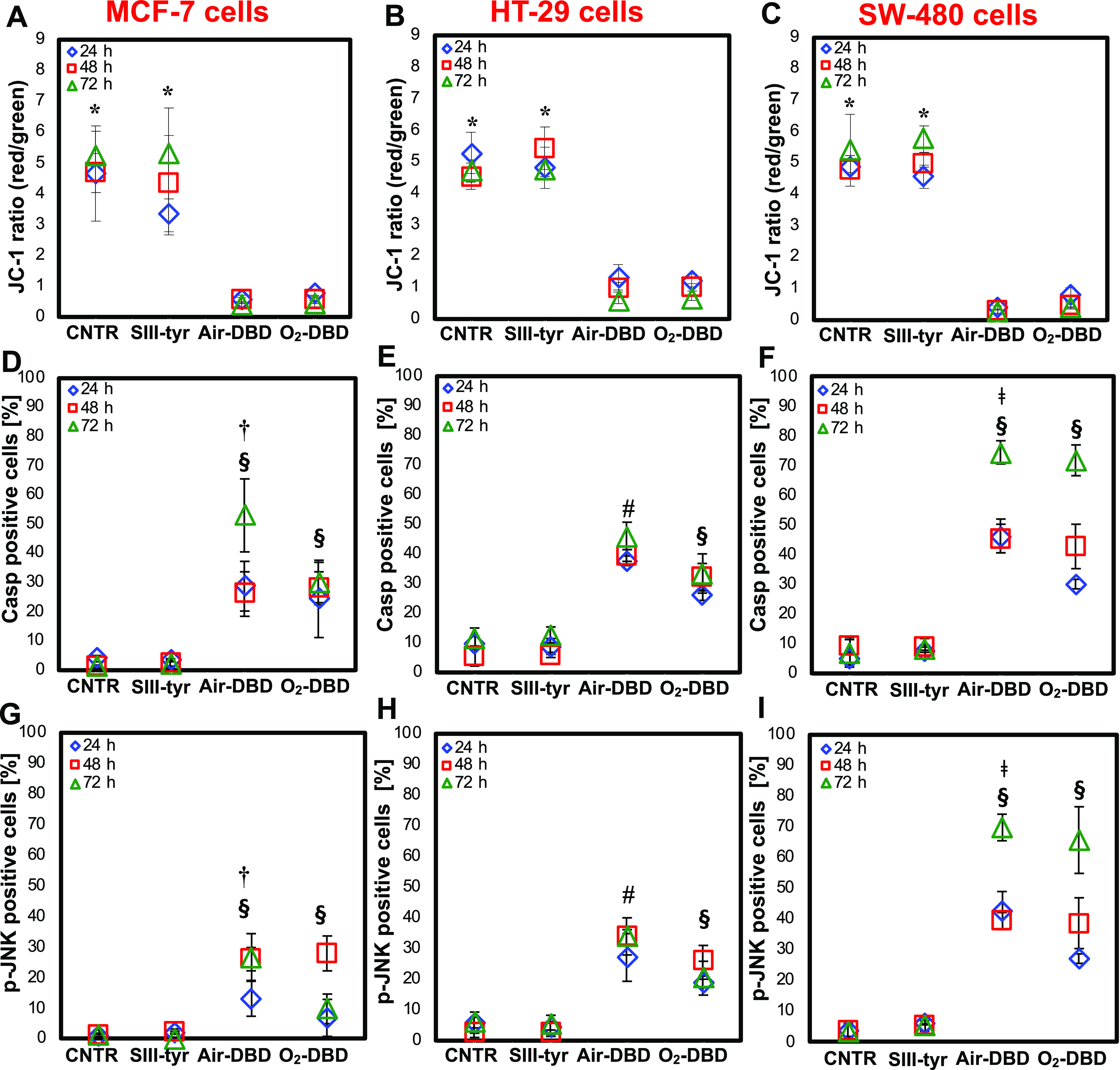
Study of mitochondrial membrane potential and activation of apoptotic markers. Pro-mitochondrial membrane potential and apoptotic effect of PTWS in MCF-7 (A, D, G), HT-29 (B, E, H), and SW-480 (C, F, I) cancer cells, after 24, 48, and 72 h of 2-h treatment with SIII-tyr, air-DBD, and O_2_-DBD. (A–C) Quantitative analysis of the fluorescence of JC-1 red and green intensity ratio. (D–I) Quantitative analysis of the activation of apoptotic markers caspase-3 (D–F) and p-JNK (G–I). The percentage of apoptotic cells was calculated by the ratio of apoptotic cells (active caspase-3-positive and p-JNK-positive) over total nuclei (DAPI-stained nuclei). The analyses on cells grown on untreated medium were used as control (CNTR). Data statistically significant according to ANOVA followed by a Bonferroni post-test (*p* < 0.05). *vs air-DBD and O_2_-DBD for each culture time; ^§^vs CNTR and SIII-tyr for each culture time; ^†^vs all treatments at 72 h; ^#^vs all treatments for each culture time; ^⧧^vs all treatments at 24 h.

In our study, the quantification of the JC-1 red/green fluorescence intensity ratio (Figure 8A–C), representative of the healthy/damaged mitochondria, reveals that the administration of air-DBD and O_2_-DBD solutions allowed a significant decrease of the MMP in all cancer cells. PTWS treatment enables the disruption of the mitochondrial membrane integrity, followed by drastic loss of membrane potential and mitochondrial dysfunction.

On the other hand, the treatment with SIII-tyr did not show apoptotic effect in any type of cancer cells analyzed, confirming that the untreated solution was almost ineffective on cells without the plasma treatment. Indeed, the JC-1 fluorescence intensity ratio (red/green) in cells exposed to SIII-tyr was very high, as in the control.

These findings highlight that both PTWS promote apoptosis via a mitochondria-dependent pathway. To further investigate the underlying molecular mechanisms of PTWS-induced apoptosis, we investigated the activation of specific signaling proteins.

Previous studies have shown that ROS activate the N-terminal c-Jun protein kinase (JNK), which subsequently phosphorylates its substrate (p-JNK) and then induces the activation of caspase-3 protein.^42^ By assuming that PTWS induce cell apoptosis by regulating the p-JNK/caspase-3 signaling pathway, we further explored PTWS’ ability in triggering the activation of p-JNK and caspase-3, which are implicated in the sequence of events leading to apoptosis.

As shown in Figure 8D–I, the treatment with PTWS significantly increased (*p* < 0.05) the levels of both cleaved caspase-3 (Figure 8D–F) and p-JNK (Figure 8G–I) in all of our cells. On the other hand, SIII-tyr did not alter the apoptotic level. Indeed, the number of cells positive for the two markers is very low and corresponds to the same positive cell count observed in the control, in all cancer cells.

Overall, the investigation of apoptotic markers indicates that both PT solutions induce cell apoptosis through a molecular mechanism involving the activation of p-JNK and caspase-3. However, PTWS evoked different responses in terms of apoptotic degree, depending on the cancer cell under investigation. Each type of cancer cells shows a different percentage of caspase-3-positive nuclei and p-JNK-positive nuclei.

The highest activation of both apoptotic markers was achieved in SW-480 cancer cells with both PTWS after 72 h of the 2-h treatment. Indeed, in these cells at that culture time, values of 74 ± 4 and 70 ± 4% after air-DBD-treatment, and of 72 ± 5 and 66 ± 11% after O_2_-DBD treatment were calculated for caspase-3 and p-JNK, respectively. Notably, the administration of both PTWS to these cancer cells induced a nearly 10-fold increase of the percentage of apoptotic positive cells when compared with the control, demonstrating their ability to trigger the execution phase of cell apoptosis.

In MCF-7 cells, after 24 and 48 h of the 2-h incubation with both air-DBD and O_2_-DBD treatment at every culture time, the percentage for both apoptotic markers was 3-fold higher than that of the control after exposure to both PTWS.

In these cancer cells, a strong activation of caspase-3 (53 ± 12%) and p-JNK (27 ± 8%) was detected 72 h after the 2-h exposure to air-DBD (Figure 8D,G). Values 5-6-fold higher than the control, and significantly higher than O_2_-DBD-induced toxicity, have been measured at each culture time.

In the case of HT-29 cells, no significant differences in apoptotic degree were observed on comparing the effects of the two PTWS (Figure 8E,H). Only a slight increase in the percentage of caspase-3-positive cells was detected again after treatment with air-DBD, especially after 72 h (46 ± 5%). The value is 4-fold higher than the control and higher than that detected at the previous culture times (24 and 48 h) with the same PT solution and with O_2_-DBD treatment, at each culture time.

Our data demonstrate that PTWS can be considered promising candidates in the research of new therapeutics for cancer treatment because they trigger apoptosis pathways that could lead to the regression of tumors.

#### 3.3.4 PTWS Affect O_2_ Consumption

To provide more evidence of PTWS’ anticancer potential, we also investigated their effect on the metabolic activity of cancer cells. As an indicator of cell metabolism performance, online oxygen concentration measurements were performed to evaluate the oxygen uptake rate of the cells 24, 48, and 72 h after the 2-h incubation in PTWS.

**Figure 9:**
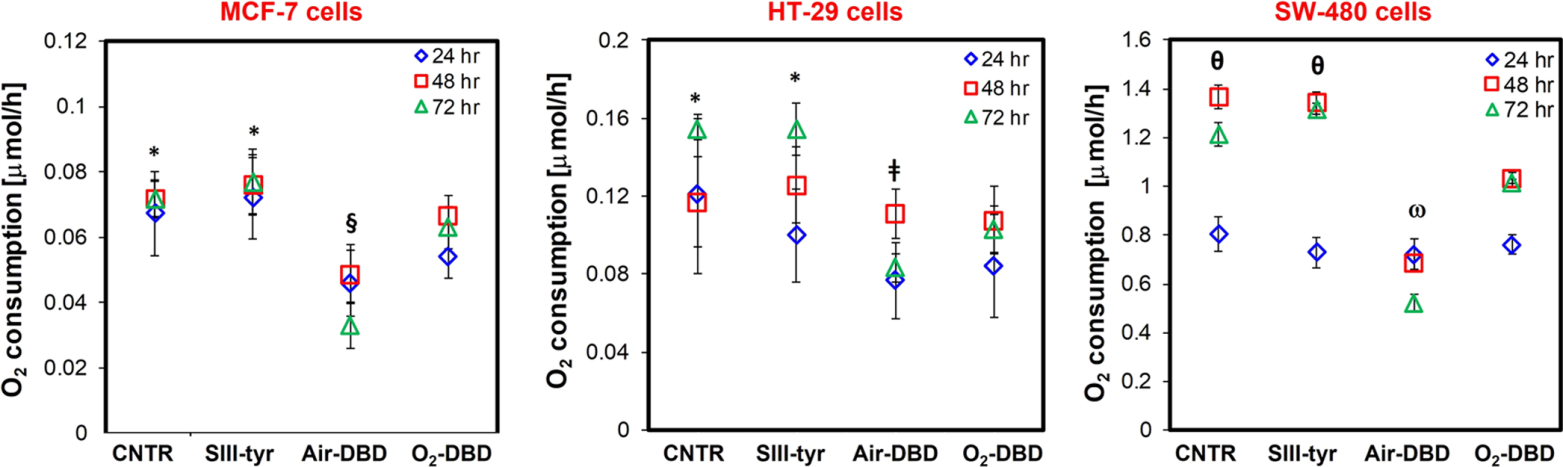
Metabolic activity. O_2_ consumption in tumor cells 24, 48, and 72 h after 2-h exposure in SIII-tyr, air-DBD, and O_2_-DBD. Data statistically significant according to ANOVA followed by Bonferroni *t*-test (*p* < 0.05). *vs air-DBD and O_2_-DBD for each culture time; ^§^vs O_2_-DBD for each culture time; ^⧧^vs O_2_-DBD at 72 h; ^θ^vs air-DBD and O_2_-DBD at 48 and 72 h; ^ω^vs and O_2_-DBD at 48 and 72 h.

As indicated in Figure 9, cells grown in untreated medium and used as control (CNTR) cultured in the FC-PLL membrane system like other samples show a balanced cell metabolism with average oxygen uptake rate values of 0.072 ± 0.005, 0.155 ± 0.006, and 1.21 ± 0.05 μmol/h for MCF-7, HT-29, and SW-480 cells, respectively, after 72 h. The rate of oxygen consumption in all cancer cells was also increased after exposure to the SIII-tyr solution, confirming its lack of toxicity.

On the contrary, as expected, according to the results on apoptotic death and intracellular ROS investigation, air-DBD and O_2_-DBD PTWS considerably brought down the oxygen uptake owing to their toxic effect. In addition, our results highlight a higher efficacy of air-DBD with respect to O_2_-DBD in reducing the oxygen uptake. Especially after 72 h, in each type of cancer cells, air-DBD caused a significant decrease of oxygen consumption to average values of 0.033 ± 0.001 μmol/h in MCF-7 cells, 0.083 ± 0.001 μmol/h in HT-29 cells, and 0.523 ± 0.034 μmol/h in SW-480 cells.

## 4 Conclusions

For this study, l-tyrosine was added to an electrolyte (SIII) solution containing citrate and acetate. The solution was plasma treated and utilized against four cancer cell lines to evaluate the combined effects of CAP-generated RONS and l-tyrosine. The generation of RONS in the SIII solution was enhanced when the treatment was performed in presence of l-tyrosine, more for H_2_O_2_ than for NO_2_^–^ ions’ production. Plasma-treated solutions containing l-tyrosine exerted high antiproliferative, pro-oxidant, and pro-apoptotic effects on the cells investigated, with a cell-dependent effect. A relevant cytotoxic activity was observed after 72 h. The neuroblastoma (SHSY-5Y) cell line resulted more sensitive to the PTWS treatment than the other cancer cells. The combination of the pro-oxidant effect of l-tyrosine and its byproducts with the RONS could therefore represent a concrete way to synergistically attack tumor cells with PTWS components. Altogether, our findings show that a significant increase of ROS formation in all cancer cells investigated occurred after PTWS treatment, indicating a pro-oxidant-mediated cytotoxic effect. This was accompanied by a loss of mitochondria membrane potential, suggesting that PTWS-mediated apoptosis may be related to the activation of the mitochondrial-mediated pathway. In addition, our findings also suggest that the molecular mechanism of the pro-apoptotic effect involves the activation of the p-JNK/caspase-3 signaling pathway. It was also found that PTWS administration influences the metabolic activity of cancer cells, as shown by the oxygen uptake results.

The effects induced by solutions exposed to air-DBD were revealed to be more cytotoxic than that exposed to O_2_-DBD, probably depending on their different ROS/RNS production ratios and on the different organic derivatives produced, as confirmed by the spectrophotometric data.

## Data Availability

The authors declare that the data supporting the findings of this study are available within the manuscript. All other data are available from the corresponding author upon reasonable request.

## Supporting Information Available

Measure of pH of untreated and plasma-treated solutions; ageing of plasma-treated solutions; LC-MS of PT-tyr solution in double-distilled water and assessment of the efficacy of Tyrosine in promoting cell death (PDF)

## Abbreviations

CAP: cold atmospheric plasma
RONS: reactive oxygen and nitrogen species
ROS: reactive oxygen species
RNS: reactive nitrogen species
PTWS: plasma-treated water solutions
DBD: dielectric barrier discharge
DBDs: dielectric barrier discharges
LOD: limit of detection
SIII: electrolyte rehydrating III solution
PT-SIII: plasma-treated electrolyte rehydrating III solution
SIII-tyr: electrolyte rehydrating III solution containing tyrosine
PT-SIII-tyr: plasma-treated electrolyte rehydrating III solution containing tyrosine
O_2_-DBD: dielectric barrier discharge fed with oxygen and PTWS treated with it
Air-DBD: dielectric barrier discharge fed with air and PTWS treated with it
CNTR: untreated cell culture medium used as control
DCF: 2′,7′-dichlorofluorescein
H_2_DCF-DA: 2′,7′-dichlorodihydrofluorescein diacetate
MMP: mitochondria membrane potential
JNK: N-terminal c-Jun protein kinase
p-JNK: phosphorylated N-terminal c-Jun protein kinase
FC-PLL: fluorocarbon membranes coated with poly-l-lysine
MTT: 3-(4,5-dimethylthiazol-2-yl)-2,5-diphenyltetrazolium bromide
LSCM: laser scanning confocal microscope
JC-1: tetraethyl-benzimidazolyl-carbocyanine iodide
SDR: sensor dish reader
