# Supplementary material for: Anticancer Effects of Plasma-Treated Water Solutions from Clinically Approved Infusion Liquids Supplemented with Organic Molecules
Source: ACS Omega. 2023 Sep 1;8(37):33723–36. doi: 10.1021/acsomega.3c04061 (PMC10515361; doi:10.1021/acsomega.3c04061)
Supplement: Supplementary file 1 — ao3c04061_si_001.pdf [file ao3c04061_si_001.pdf]

Supporting Information

ANTICANCER EFFECTS OF PLASMA TREATED WATER SOLUTIONS FROM CLINICALLY APPROVED INFUSION LIQUIDS SUPPLEMENTED WITH ORGANIC MOLECULES

Valeria Veronico, [1,^] Sabrina Morelli,[2,^] Antonella Piscioneri,[2] Roberto Gristina,[3] Michele Casiello, [1] Pietro Favia,[1,3] Vincenza Armenise[1], Francesco Fracassi[1,3], Loredana De Bartolo[2,*], Eloisa Sardella[3,*]

[1] Department of Chemistry, University of Bari Aldo Moro, via Orabona, 4, 70126, Bari, Italy

[2] CNR-Institute on Membrane Technology (CNR-ITM), Via Pietro Bucci cubo 17/C, 87036, Rende (CS), Italy

[3] CNR-Institute of Nanotechnology (CNR-NANOTEC) Via Amendola, 122/D, Bari 70124, Bari, Italy

^These authors contributed equally

* Corresponding authors:
Eloisa Sardella
CNR, Institute of Nanotechnology (CNR-NANOTEC)
c/o Dept. Chemistry, University of Bari
via Orabona, 4
70126 Bari, Italy
Tel.: +39-080-5442295
Fax.: +39-0805443405
Email: eloisa.sardella@cnr.it

Loredana De Bartolo
CNR-Institute on Membrane Technology (CNR-ITM)
Via Pietro Bucci cubo 17/C
87036, Rende (CS), Italy
Tel: (+39) 0984 492036
Email: loredana.debartolo@cnr.it

Measure of pH of untreated and plasma treated solutions

The pH of PTWS remains unaltered respect to the untreated SIII-tyr solution whatever plasma treatment used (Figure S1).

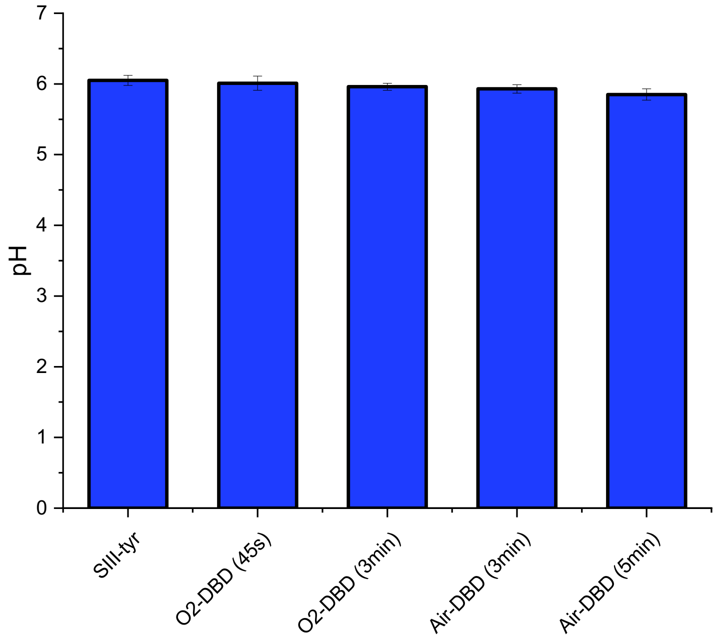

Figure S1. Acid/base character of the solutions. Measurements of pH in PTWS generated from SIII-tyrosine solutions after different DBDs ignited at 13.5 kV, 25% DC, 6 kHz, 0.5 slm flow rate on 2 ml of liquid 3 mm far from the discharge, with different gas feeds (O_2 or air) and for different treatment times (45 – 300 s).

Ageing of plasma treated solutions

The chemical composition of PTWS remains unaltered after 15 days storage at 25°C whatever plasma treatment used (Figure S2).

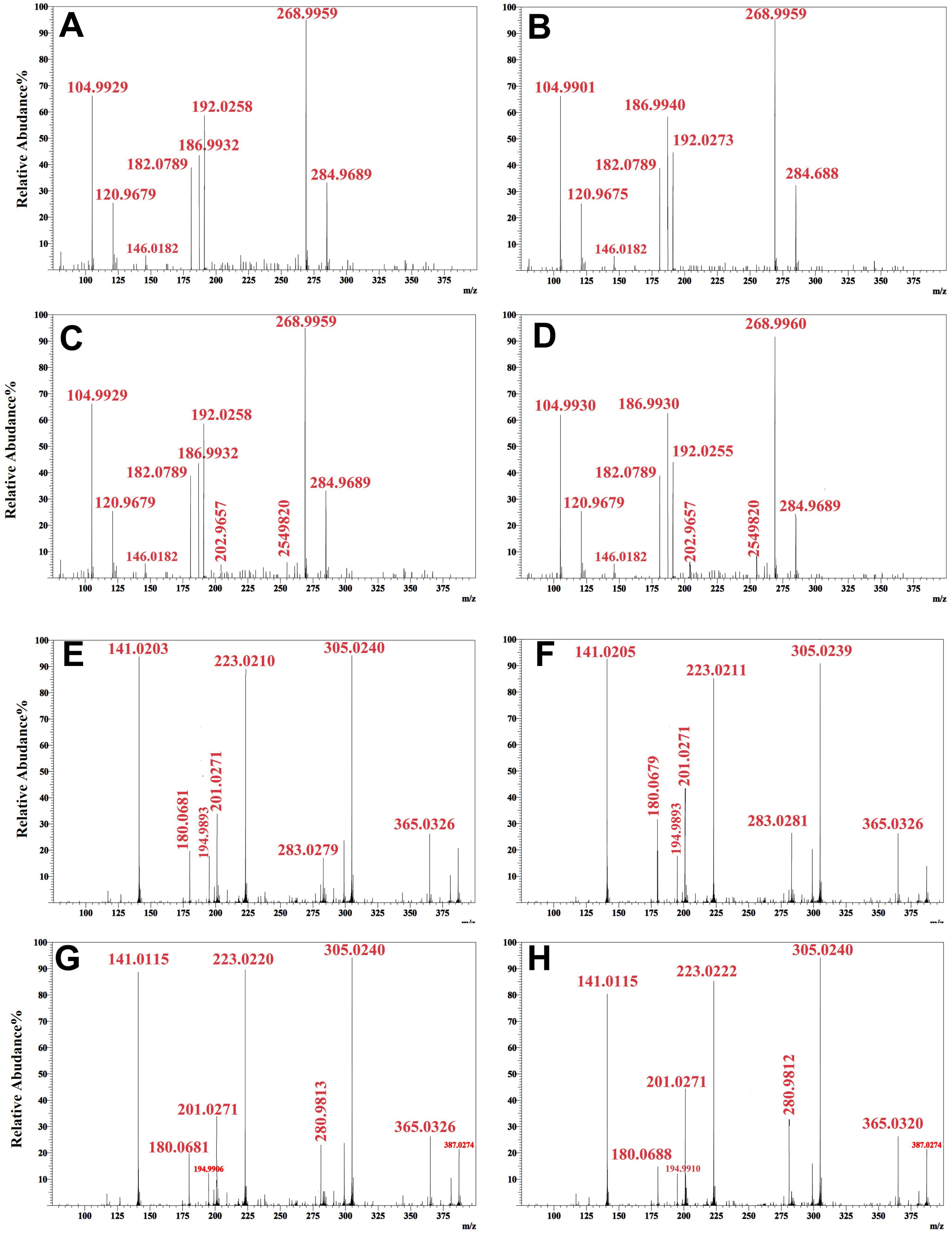

Figure S2. Ageing studied by LC-MS of PT-SIII-tyr solutions. Positive (A-B-C-D) and negative (E-F-G-H) mode spectra acquired for plasma treated in O_2 (A-B-E-F) and Air (C-D-G-H) before (A-C-E-G) and after (B-D-F-H) ageing at 25°C for 15 days.

LC-MS of PT-tyr solution in double distilled water

LC-MS characterization of plasma treated tyrosine solution in double distilled water reveals the presence of nitration and oxidation products of tyrosine depending on the gas feed used.

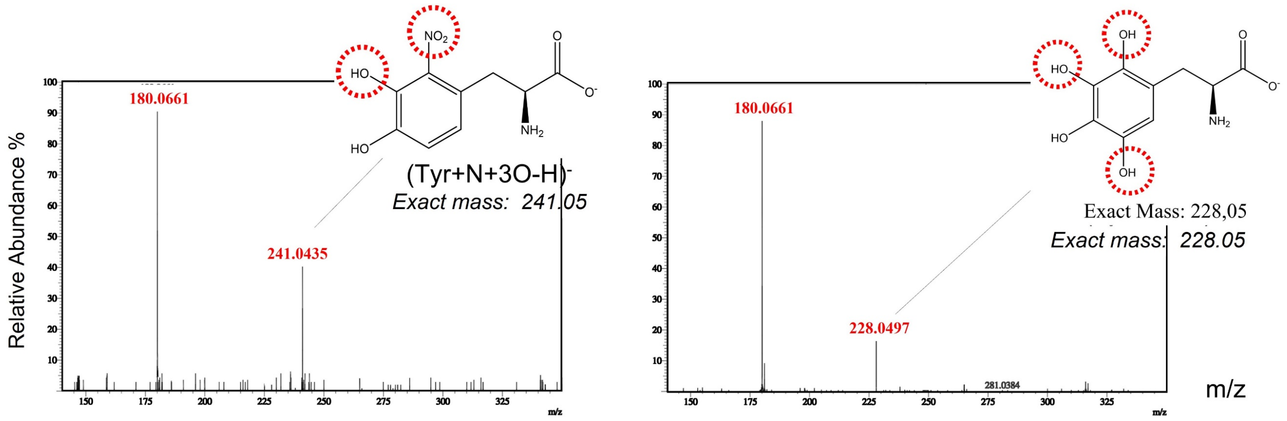

Figure S3. Oxidation and nitration products PT-tyr solutions in double distilled water. Negative mode spectra acquired for plasma treated in Air 3 min (left) and O_2 45 s (right).

Assessment of Tyrosine efficacy in promoting cell death

A first preliminary cell culture experiment, aimed at verifying the importance of the Tyrosine in the SIII treated solution to be used in plasma treatment to achieve an effect on cell lines, has been performed on SH SY5Y and HT-29 cell lines.

PTWS were prepared in the same experimental conditions used in the experiments shown in the paper with O_2 as gas feed, using SIII or SIII+tyr as liquid.

Cells were incubated for 2 hrs with Sol III O_2 45 s or with Sol III Tyr O_2 45 s solutions. After replacing the PTWS with complete medium, cells were let grown for further 24 hrs.

The obtained results, as shown in Figure S2 and S3, confirmed the importance of the presence of Tyr to achieve a reduction in cell growth.

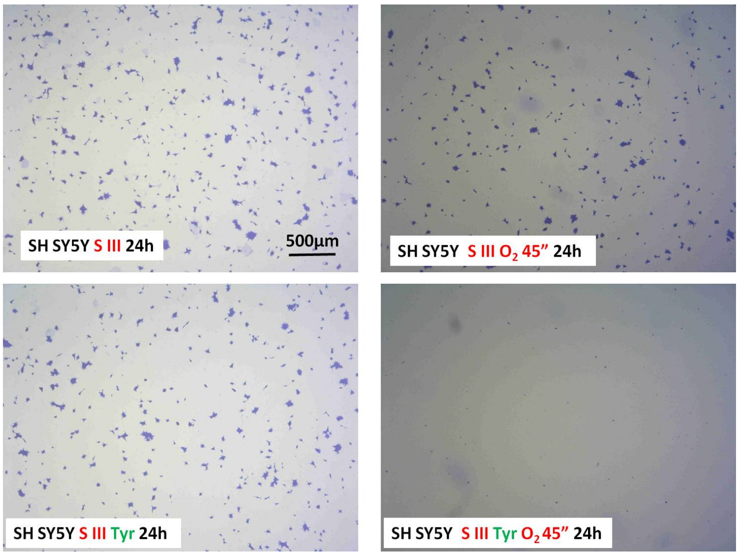

Figure S4: Deleterius effect of PT-SIII-tyr solutions toward SHSY5Y cells. Comassie Blue staining of SHSY5Y cells incubated for 2 hours to untreated and plasma treated SIII and SIII-Tyr with O_2 for 45 s analysed after 24 hours of growth.

For the HT-29 cell line since there was slight effect for PTWS obtained by using O_2 for 45 s a longer time treatment time was applied (5min plasma treatment).

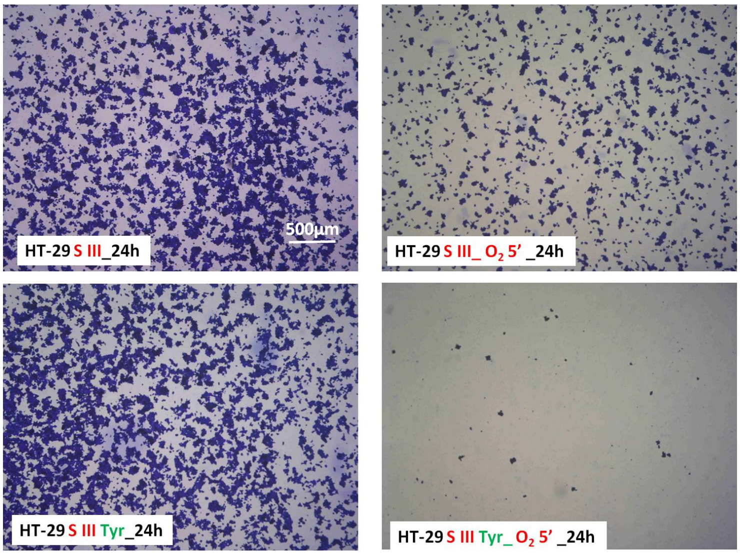

Figure S5: Deleterious effect of PT-SIII-tyr solutions toward HT29 cells. Comassie Blue staining of HT29 cells incubated for 2 hours to untreated and plasma treated SIII and SIII-Tyr with O_2 for 5 min and analysed after 24 hours of growth.
